# Defining Hypo-Methylated Regions of Stem Cell-Specific Promoters in Human iPS Cells Derived from Extra-Embryonic Amnions and Lung Fibroblasts

**DOI:** 10.1371/journal.pone.0013017

**Published:** 2010-09-27

**Authors:** Koichiro Nishino, Masashi Toyoda, Mayu Yamazaki-Inoue, Hatsune Makino, Yoshihiro Fukawatase, Emi Chikazawa, Yoriko Takahashi, Yoshitaka Miyagawa, Hajime Okita, Nobutaka Kiyokawa, Hidenori Akutsu, Akihiro Umezawa

**Affiliations:** 1 Department of Reproductive Biology, National Institute for Child Health and Development, Tokyo, Japan; 2 Department of Developmental Biology, National Institute for Child Health and Development, Tokyo, Japan; RIKEN Brain Science Institution, Japan

## Abstract

**Background:**

Human induced pluripotent stem (iPS) cells are currently used as powerful resources in regenerative medicine. During very early developmental stages, DNA methylation decreases to an overall low level at the blastocyst stage, from which embryonic stem cells are derived.Therefore, pluripotent stem cells, such as ES and iPS cells, are considered to have hypo-methylated status compared to differentiated cells. However, epigenetic mechanisms of “stemness” remain unknown in iPS cells derived from extra-embryonic and embryonic cells.

**Methodology/Principal Findings:**

We examined genome-wide DNA methylation (24,949 CpG sites covering 1,3862 genes, mostly selected from promoter regions) with six human iPS cell lines derived from human amniotic cells and fetal lung fibroblasts as well as two human ES cell lines, and eight human differentiated cell lines using Illumina's Infinium HumanMethylation27. A considerable fraction (807 sites) exhibited a distinct difference in the methylation level between the iPS/ES cells and differentiated cells, with 87.6% hyper-methylation seen in iPS/ES cells. However, a limited fraction of CpG sites with hypo-methylation was found in promoters of genes encoding transcription factors. Thus, a group of genes becomes active through a decrease of methylation in their promoters. Twenty-three genes including *SOX15*, *SALL4*, *TDGF1*, *PPP1R16B* and *SOX10* as well as *POU5F1* were defined as genes with hypo-methylated SS-DMR (Stem cell-Specific Differentially Methylated Region) and highly expression in iPS/ES cells.

**Conclusions/Significance:**

We show that DNA methylation profile of human amniotic iPS cells as well as fibroblast iPS cells, and defined the SS-DMRs. Knowledge of epigenetic information across iPS cells derived from different cell types can be used as a signature for “stemness” and may allow us to screen for optimum iPS/ES cells and to validate and monitor iPS/ES cell derivatives for human therapeutic applications.

## Introduction

Human embryonic stem (ES) cells [1] and induced pluripotent stem (iPS) cells [2], [3], [4], [5] are currently used as powerful resources in regenerative medicine. However, epigenetic mechanisms of “stemness” remain unknown. DNA methylation is known to be a key component in normal differentiation and development [6], [7]. Tissue-specific genes, such as *OCT-4/3* [8], *Sry* (sex determining region on Y chromosome) [9] and *MyoD* [10], show tissue-specific demethylation corresponding to their expression during development. Furthermore, DNA methylation in cells specifically varies depending on cell lineage and tissue types [7]. Transformation to iPS cells from differentiated cells requires a process of epigenetic reprogramming [11]. Understanding the epigenetic regulation in human pluripotent stem cells, therefore, enable us to elucidate “stemness” and to screen for optimum iPS/ES cells for human therapeutic applications. Human extra-embryonic amnion cells are a useful cell source for generation of iPS cells, because they can be collected without invasion and are conventionally freeze-storable. Recently, we generated iPS cells from human amnion cells as well as human fetal lung fibroblast cells [12], [13]. Here, we show DNA methylation profiles of human pluripotent stem cells including iPS cells, which were derived from extra-embryonic amnion cells and fetal lung fibroblast cells, and human ES cells. We also defined another subset that may play a key practical role in maintaining the state of “stemness”.

## Results

### Analysis of genome-wide DNA methylation

Human iPS cell lines (MRC-iPS [13] and AM-iPS cell lines [12]) independently established in our laboratory by retroviral infection of 4 genes (*OCT-3/4*, *SOX2*, *c-MYC*, and *KLF4*), based on the Yamanaka's pioneer protocols [2] from 2 fully differentiated cells (MRC-5, fetal lung fibroblast cells, and AM936EP, amnion cells), were used as a primary source for experimentation (Table 1). These cells clearly showed human iPS characters [12], [13].

**Table 1:** A list of human cells analyzed for a methylation state in this study.

Cell ID	Description	ability of differentiation
MRC5	Fetal lung fibroblast cells	None
MRC-iPS-11	MRC5-derived iPS cells (P4)	Pluripotent
MRC-iPS-19	MRC5-derived iPS cells (P4)	Pluripotent
MRC-iPS-75	MRC5-derived iPS cells (P4)	Pluripotent
AM936EP	Amnion-derived cells (P6)	None
AM-iPS-3	AM936EP -derived iPS cells (P4)	Pluripotent
AM-iPS-6	AM936EP -derived iPS cells (P4)	Pluripotent
AM-iPS-8	AM936EP -derived iPS cells (P4)	Pluripotent
UtE1104	Endometrium-derived cells (P7)	None
H4-1	Bone marrow stroma-derived cells (P26)	None
Mim1508E	Auricular cartilage-derived cells (P1)	Cartilage
Yub636BM	Extra finger bone marrow-derived cells (P3)	Bone
PAE551	Placental artery endothelial cells (P13)	None
Edom22	Menstrual blood-derived cells (P1)	Myoblast
HUES3	Embryonic stem cells (P29)	Pluripotent
HUES8	Embryonic stem cells (P24)	Pluripotent

Numbers in parenthesis with P indicate passage in culture on the cells used in the methylation analysis.

**Figure 1:**
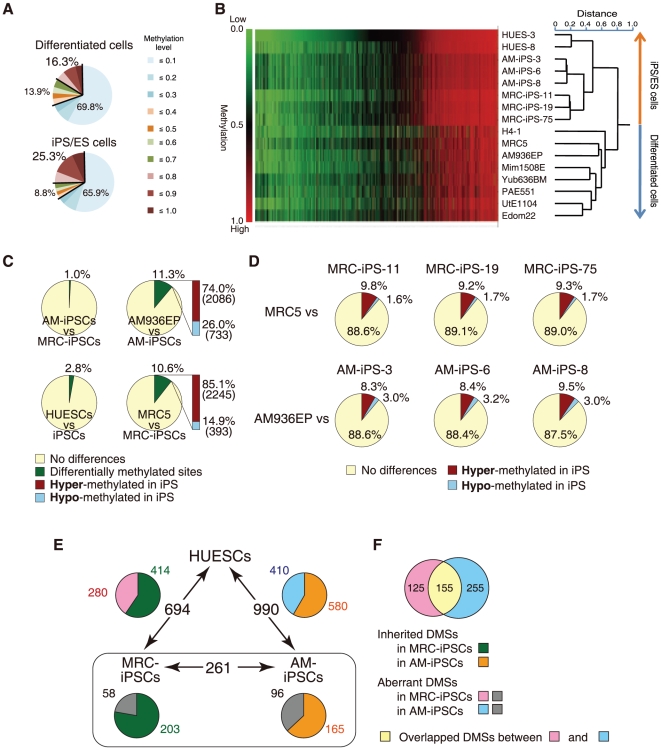
The ratio of hyper-methylated sites in iPS/ES cells was significantly larger than that of the differentiated cells. (**A**) Ratio of Low (methylation score≤0.3), Middle (0.3<score≤0.7), and High (0.7<score) methylated states in 24,949 CpG sites. (**B**) Clustering analysis. Heat map showing hyper-methylation in human iPS/ES cells compared with differentiated cells. The Heat map in hierarchical clustering analysis represented DNA methylation levels from completely methylated (red) to unmethylated (green). Epigenetic distances (Euclidean Distance) were calculated by NIA Array. (**C**) Comparisons of CpG sites between two groups show high similarities between AM-iPS and MRC-iPS cells or between human ES cells (HUESCs) and iPS cells (iPSCs). In contrast, 11.3% and 10.6% of CpG sites are differentially methylated in AM-iPS and MRC-iPS cells, respectively, compared to their parental cells (AM936EP and MRC5). It should be noted that 74.0% and 85.1% of the differentially methylated sites (DMSs) are hyper-methylated in AM-iPS and MRC-iPS cells, respectively, compared to their parental cells. (**D**) Comparison of the 24,949 CpG sites between iPS cells and their parental cells. (**E**) DMSs among human ES cells, AM- and MRC-iPS cells. The relative amount of inherited/aberrant DMSs is indicated in the pie chart. (**F**) Overlapped aberrant DMSs between MRC- and AM-iPS cells.

To examine DNA methylation status in six iPS, two ES [14], and eight differentiated cell lines (Table 1), we therefore examined genome-wide DNA methylation using Illumina's Infinium HumanMethylation27 BeadChip, on which oligonucleotides for 27,578 CpG sites covering more than 14,000 genes are mounted, mostly selected from promoter regions. This assay system provides advantageous quantitative measurement. DNA methylation levels were recorded using a scoring system ranging from “0” (completely unmethylated) to “1” (fully-methylated). Using multiple repetitions, we analyzed 24,949 out of 27,578 CpG sites with 16 samples (see Materials and Methods), categorizing them into three groups; Low (score≤0.3), Middle (0.3<score≤0.7), or High (0.7<score) methylation. Overall, methylation levels in pluripotent stem cells and differentiated cells are shown in Fig. 1A, with the levels in each cell line presented in Table S1. While the percentage of the High class in differentiated cells was 16.3% on average, the percentage in iPS/ES cells was 25.3% (Fig. 1A). The number of CpG sites categorized in the High class is significantly greater in pluripotent stem cells compared with differentiated cells. Hierarchical clustering analysis clearly discriminates iPS/ES cells from the differentiated cells (Fig. 1B). Hyper-methylated sites (shown in red) are widespread in the heat map in iPS/ES cells, compared with the differentiated cells (Fig. 1B), suggesting that gene promoters in iPS/ES cells are hyper-methylated, compared with those in differentiated cells.

About two-thirds of the CpG sites were at a Low methylated level in both iPS/ES cell and differentiated cell groups (Fig. 1A and B). Another computation found 13,971 CpG sites to consistently show a score of lower than 0.3. This suggests that a significant fraction of the CpG sites examined may have less involvement in methylation, although some might become methylated under different conditions. As most CpG sites on the chip were chosen simply based on the location in promoters, it is possible that some CpG sites may be positioned at a distance from the target site, even in a promoter controlled by DNA methylation. Analysis of our and all published data indicated that a group of CpG sites more suitable to methylation analyses could be identified, allowing us to focus attention on specific changes in methylation levels seen between iPS/ES and differentiated cells.

#### Differentially methylated site (DMS) in the promoters

Firstly, we defined the “differentially methylated site” (DMS), representing a CpG site whose score differed 0.3 points and more between the two cell groups. The DMSs between MRC-iPS and AM-iPS cells, and also between iPS and ES cells, were only 1.0% and 2.8% of all the CpG sites, respectively (Fig. 1C), suggesting that iPS and ES cells have similar methylation status. In contrast, the DMSs between AM936EP and AM-iPS cells, and between MRC-5 and MRC-iPS cells, were 11.3% and 10.6%, respectively, suggesting that iPS cells and their parental cells have differentially methylated status (Fig. 1C and D). It should be noted that approximately 80% of the DMSs between the iPS cells and their parental cells changed to a “hyper-methylated” state from a “hypo-methylated” state in iPS cells (Fig. 1C). Comparison of DMSs between AM- and MRC-iPS cells, and between iPS and ES cells show slight but significant difference (Fig. 1C). In 261 DMSs between MRC- and AM-iPS cells (MA-DMSs), 203 sites in AM-iPS cells and 165 in MRC-iPS cells showed no difference from their parental cells, suggesting that these sites in iPS cells are inherited from their tissue origin (Fig. 1E). In addition, 414 out of 694 DMSs between MRC-iPS and ES cells (ME-DMSs) and 581 out of 990 DMSs between AM-iPS and ES cells (AE-DMSs) are inherited DMSs (Fig. 1E). Interestingly, approximately 40% of DMSs between iPS and ES cells are iPS-specific DMSs, meaning that these sites are aberrant methylated in iPS cells (Fig. 1E). In aberrant DMSs, 155 sites overlapped between MRC- and AM-iPS cells (Fig. 1F and data set S1, S2, S3). Overlapping aberrant DMSs are located at promoters in genes such as gene for FZD10, MMP9 and three zinc finger proteins (ZNF551, ZNF513 and ZNF540). These genes are hyper-methylated in iPS cells than parental cells and ES cells. Approximately 80% of aberrant DMSs are hyper-methylated, compared with parent cells and ES cells.

#### Defining stem cell specific differentially methylated regions (SS-DMRs)

**Figure 2:**
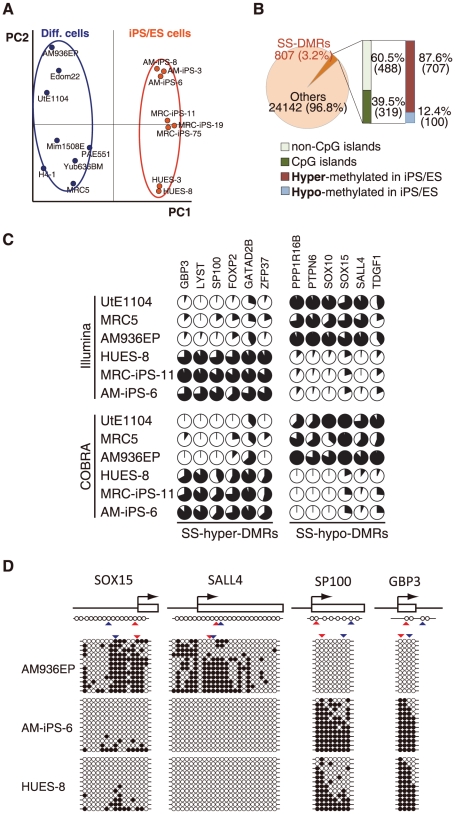
Pluripotent stem cells are significantly more hyper-methylated than differentiated cells. (**A**) Principal component analysis (PCA) for DNA methylation states of 24,949 CpG sites with 16 human cell lines. The PC1 axis clearly distinguish iPS/ES cell group from differentiated cells, while human iPS cells are very close to human ES cells. (**B**) Stem cell-specific differently methylated regions (SS-DMRs) were defined by PC1. In the pluripotent stem cells, 60.5% of the SS-DMRs are located outside of CpG islands and 87.6% of the SS-DMRs are hyper-methylated. (**C**) DNA methylation levels at promoter regions in 12 representative genes determined by Illumina Infinium HumanMethylation27 assay and Bio-COBRA. Details of these genes are described in Table S6B. The promoter regions of these genes were defined as the SS-DMRs. The relative amount of methylated DNA ratio is indicated as the black area in the pie chart. The same methylation patterns in 12 regions were detected both by Infinium assay and COBRA. (**D**) Bisulfite sequencing analysis of the same regions that were analyzed by Infinium assay and COBRA assay in *SOX15*, *SALL4*, *SP100* and *GBP3*. (Top) Schematic diagram of the genes. Arrows, open boxes and open circles represent transcription start site, first exon and position of CpG sites, respectively. (Bottom) Open and closed circles indicate unmethylated and methylated states, respectively. Red and blue arrowheads represent the position of CpG sites in Infinium assay and COBRA, respectively.

Principal component analysis (PCA) shows high similarity among human iPS and ES cells and clearly separates the iPS/ES cells from the differentiated cells, which is supported by hierarchical clustering analysis (Fig. 1B and Fig. 2A). Based on principal component 1 (PC1), 807 (3.2%) out of 24,949 sites were deduced to change their methylation state along with “stemness” (Fig. 2B). We designated a region represented by such CpG sites as “stem cell specific differentially methylated regions” (SS-DMRs). Of the 807 SS-DMRs, 39.5% (319 sites) are localized on CpG islands, whereas 60.5% (488 sites) are not (Fig. 2B), although 72.5% CpG sites on the bead-chips occur on CpG islands. Thus, promoter regions on non-CpG islands were more affected during reprogramming towards pluripotent stem cells. 707 sites (87.6%) of the SS-DMRs were significantly increased in the methylation levels in iPS/ES cells, compared with those in the differentiated cells, and we designated these sites as “stem cell specific hyper-differentially methylated regions (SS-hyper-DMRs) ” (Fig. 2B and data set S4). In contrast, 100 sites (12.4%) were decreased and designated as “stem cell specific hypo-differentially methylated regions (SS-hypo-DMRs) ” (Fig. 2B and data set S5). We also confirmed the methylation state in the promoter regions for some of the detected genes by another means, i.e. quantitative combined bisulfite restriction analysis (COBRA) [15] (Fig. 2C). In addition, results of bisulfite sequencing of the region surrounding the SS-DMRs corresponded to results of Infinium assay and COBRA (Fig. 2D).

#### Gene ontology analysis with the SS-DMRs

We searched gene ontology databases for details of the SS-DMRs. Interestingly, SS-hypo-DMRs are abundant in genes related to nucleic acid binding and transcription factors, which may function in iPS cells. On the other hands, SS-hyper-DMRs are abundant in genes related to differentiation (Table 2). We also subjected the SS-DMRs to KEGG (Kyoto Encyclopedia of Genes and Genomes) pathway. Cytokine receptor interaction cascade, MAPK signaling, and Neuroactive ligand-receptor interaction are all major keywords for SS-hyper-DMRs (Table S2).

**Table 2:** A list of top 7 categories of GO Term in “SS-DMRs”.

Molecular Function		
PantherID: GO Term	Count. Genes	%
**SS-hypo-DMRs**		
MF00042:Nucleic acid binding	30	41.10%
MF00036:Transcription factor	15	20.55%
MF00099:Small GTPase	11	15.07%
MF00137:Glycosyltransferase	8	10.96%
MF00082:Transporter	6	8.22%
MF00154:Metalloprotease	6	8.22%
MF00098:Large G-protein	6	8.22%
**SS-hyper-DMRs**		
MF00213:Non-receptor serine/threonine protein kinase	124	20.98%
MF00262:Non-motor actin binding protein	119	20.14%
MF00001:Receptor	80	13.54%
MF00131:Transferase	76	12.86%
MF00099:Small GTPase	66	11.17%
MF00242:RNA helicase	57	9.64%
MF00261:Actin binding cytoskeletal protein	53	8.97%

#### Expression of genes with SS-DMRs in human iPS/ES cells

**Figure 3:**
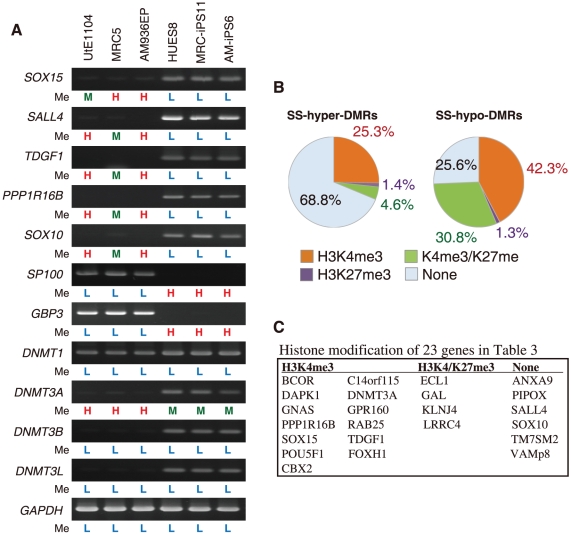
Expression and histone modification of the SS-DMRs related genes. (**A**) Expression patterns of representative genes. RT-PCR analysis of 7 representative genes and methyltransferase genes. Methylation levels (Me) of each promoter are shown under each panel. H = High methylation (0.7<score); M = Middle methylation (0.3<score≤0.7); L = Low methylation (score≤0.3). (**B**) Comparable distribution of the SS-DMR and histone trimethylation (me3) of H3K4 and H3K27. Percentage of H3K4me3, H3K27me3, bivalent H3K4me3/K27me3 or non-modification on genes in SS-hyper-DMRs and in SS-hypo-DMRs. (**C**) Histone modification of 23 genes in Table 3.

To address whether changes in DNA methylation state are associated with expression levels, we surveyed genes showing more than 5-fold change of expression in human iPS/ES cells, compared with those in differentiated cells, using the GEO database [16], [17]. Twenty-three genes represented by SS-hypo-DMRs were found in “genes significantly expressed in iPS/ES cells” (Table 3 and Table S3A). Representative genes, including *SOX15*, *SALL4*, *TDGF1*, *PPP1R16B* and *SOX10*, are expressed with hypo-methylation states in iPS/ES cells (Fig. 3A). On the other hand, forty-three genes represented by SS-hyper-DMRs were found in “genes significantly suppressed in iPS/ES cells” (Table S3B and S4). Representative genes, *SP100* and *GBP3*, are suppressed by hyper-methylation in iPS/ES cells (Fig. 3A). Among DNA methyltransferases, *DNMT3B* was reported to be highly expressed in human ES cells [18]. *DNMT3A*, *DNMT3B* and *DNMT3L* were indeed expressed in iPS/ES cells (Fig. 3A). The *DNMT3A* promoter in iPS/ES cells became demethylated, while *DNMT3B* and *DNMT3L* promoters remained low methylated during reprogramming (Fig. 3A and Table S5A), leading us to analyze chromatin in iPS/ES cells in addition to DNA methylation.

**Table 3:** A list of 23 genes with SS-hypo-DMRs exhibiting ‘high’ expression in human iPS/ES cells.

TargetID	Gene name	Fold change of expression	DNA methylation level in iPS/ES cells	DNA methylation level in Diff. cells
cg07337598	*ANXA9*, *annexin A9*	5.53	0.294±0.023	0.712±0.014
cg24183173	*BCOR*, *BCL-6 interacting corepressor*	5.06	0.014±0.005	0.784±0.051
cg21207436	*C14orf115*, *hypothetical protein LOC55237*	63.49	0.052±0.005	0.442±0.036
cg22892904	*CBX2*, *chromobox homolog 2*	11.48	0.068±0.006	0.607±0.051
cg24754277	*DAPK1*, *death-associated protein kinase 1*	28.34	0.115±0.005	0.708±0.049
cg21629895	*DNMT3A*, *DNA cytosine methyltransferase 3 alpha*	12.88	0.452±0.011	0.769±0.039
cg02932167	*ECEL1*, *endothelin converting enzyme-like 1*	17.57	0.115±0.007	0.672±0.059
cg25431974	*ECEL1*, *endothelin converting enzyme-like 1*	17.57	0.125±0.013	0.674±0.093
cg04515567	*FOXH1*, *forkhead box H1*	55.88	0.602±0.014	0.855±0.006
cg04464446	*GAL*, *galanin preproprotein*	194.63	0.241±0.022	0.735±0.056
cg00943909	*GNAS*, *guanine nucleotide binding protein*	47.33	0.076±0.016	0.528±0.081
cg27661264	*GNAS*, *guanine nucleotide binding protein*	47.33	0.037±0.005	0.355±0.054
cg18741908	*GPR160*, *G protein-coupled receptor 160*	60.48	0.068±0.006	0.466±0.038
cg20674521	*KCNJ4*, *potassium inwardly-rectifying channel J4*	6.11	0.306±0.024	0.772±0.043
cg21129531	*LRRC4*, *netrin-G1 ligand*	7.04	0.027±0.004	0.788±0.058
cg06144905	*PIPOX*, *L-pipecolic acid oxidase*	42.97	0.100±0.015	0.558±0.080
cg13083810	*POU5F1*, *POU domain*; *class 5*;	559.14	0.563±0.025	0.919±0.009
cg27377213	*PPP1R16B*, *protein phosphatase 1 regulatory inhibitor subunit 16B*	65.86	0.097±0.009	0.796±0.102
cg19580810	*RAB25*, *member RAS oncogene family*	6.16	0.062±0.010	0.703±0.030
cg09243900	*RAB25*, *member RAS oncogene family*	6.16	0.105±0.013	0.595±0.031
cg06303238	*SALL4*, *sal-like 4*	227.35	0.013±0.005	0.736±0.075
cg06614002	*SOX10*, *SRY-box 10*	5.23	0.028±0.005	0.829±0.046
cg01029592	*SOX15*, *SRY-box 15*	10.19	0.174±0.011	0.692±0.032
cg10242476	*TDGF1*, *teratocarcinoma-derived growth factor 1*	2472.59	0.146±0.013	0.387±0.052
cg20277416	*TM7SF2*, *transmembrane 7 superfamily member 2*	5.23	0.380±0.017	0.833±0.027
cg05656364	*VAMP8*, *vesicle-associated membrane protein 8*	9.69	0.070±0.010	0.698±0.081

Fold change of expression: Fold change of expression of the listed gene in human iPS/ES cells against the expression level in differentiated cells.

#### Histone H3K4 and H3K27 modification of genes with the SS-DMRs

Histone modification is another important mechanism in epigenetics. Methylation of lysine 4 (K4) and 27 (K27) on histone H3 is associated with active and silent gene expression, respectively [19], while bivalent trimethylation (me3) of H3K4 and K27 represses their gene expression in ES cells [20], [21]. Based on the database of the UCSC Genome Bioinformatics, the promoter of *DNMT3B* in human ES cells is highly modified by 3K4me3, compared with that in human lung fibroblasts (Table S5B). No differences in histone modification of H3K4me3 or H3K27me3 between ES and lung fibroblasts at promoter of *DNMT3L* were detected (Table S5B). We also compared DNA methylation of the SS-DMRs with reported data for whole-genome mapping of H3K4me3 and H3K27me3 in the promoter regions of human ES cells [22]. In SS-hyper-DMRs, 68.8% do not have trimethylation of H3K4 and K27 (Fig. 3B). On the other hand, 42.3%, 1.3%, and 30.8% of SS-hypo-DMRs are marked with H3K4me3, H3K27me3, and bivalent H3K4me3 and K27me3, respectively (Fig. 3B). Thirteen out of the 23 genes in Table 3 have trimethylation solely on K4 (Fig. 3C). Six genes have no histone trimethylation on K4 and K27 and the rest have bivalent K4/K27 trimethylation (Fig. 3C).

## Discussion

Our genome-wide DNA methylation analysis shows that iPS and ES cells have similar methylation status although DNA methylation status of AM-iPS cells was closer to that of MRC-iPS cells than to that of ES cells in a small fraction. Doi et al. reported 71 differential methylated regions covering 64 genes between human iPS cells and ES cells [23]. Comparison of 535 aberrant DMSs (overlapping, 155; MRC-iPS specific, 125; AM-iPS specific, 255) with Doi's data, six genes that are *HOXA9*, *A2BP1*, *FZD10*, *SOX2*, *PTPRT* and *HYPK* overlapped. The inconsistency of most DMSs may be due to the stochastic nature of aberrant methylation through the genome. Human iPS and ES cells have general hyper-methylated status compared with differentiated cells. Our present genome-wide study indicates that pluripotent stem cells are generally hyper-methylated at promoter regions in comparison with differentiated cells. In the SS-DMRs, the number of CpG sites on non-CpG islands is grater than those on CpG islands, suggesting that promoter regions on non-CpG islands were more affected during reprogramming towards pluripotent stem cells. This result is consistent with the suggestion by Fouse et al. (2008) [24] that DNA methylation in mouse ES cells primarily occurred on non-CpG island regions of promoters.

Gene ontology analysis shows that signal transduction and transmembrane are major keywords for SS-hyper-DMRs. Most genes with SS-hyper-DMRs relate to differentiation. Recent studies demonstrate that blocking the p53 and TGFß pathways improves efficiency of generation of iPS cells [25], [26], [27], [28], [29], [30]. Some genes related to these pathways are included in SS-hyper-DMR. Approximately 70% of SS-hyper-DMR have no modification of H3K4 and H3K27, suggesting that most genes with SS-hyper-DMRs are rigorously turned off by DNA methylation. By combining these findings with the result of *DNMT3A*, *DNMT3B* and *DNMT3L* induction in iPS/ES cells, we suggest that SS-hyper-DMRs apparently include genes that play a role in differentiated cells. Moreover, they must be silenced by DNMTs to establish pluripotency. We then identified 43 genes with SS-hyper-DMRs from “genes significantly suppressed in iPS/ES cells” (Table S3B and S4). In particular, *GBP3* and *SP100* could be used as epigenetic markers for pluripotency.

In addition, we successfully determined 23 genes with SS-hypo-DMRs from “genes significantly expressed in iPS/ES cells” (Table 3 and Table S3A). Those genes may start to be induced by demethylation and a significant subset of genes that act for de-differentiation escape methylation in pluripotent stem cells during global reprogramming. Promoters of most marker genes expressed in human iPS/ES cells were low methylated in all cells examined (Table S5C). Analysis of histone modification of H3K4me3 and K27me3 from the database suggested that expression of *DNMT3B* might be regulated by methylation of H3K4 but expression of *DNMT3L* might not be under control of histone modification of H3K4me3 and K27me3. Most genes with SS-hypo-DMRs without expression in human iPS/ES cells have modification of H3K4me, bivalent H3K4me/K27me, or none, but do not have H3K27me3 modification. These genes may therefore be ready to be activated upon differentiation.

These findings are in generally consistent with the previous reports that have compared methylation profiles in somatic cells, iPS cells, and ES cells [23], [31], [32]. However, their analyses were limited only to human fibroblasts as a source for generation of iPS cells. In this study, we analyzed human extra-embryonic amnion cells and iPS cells. The DNA methylation profile at promoter sites clearly distinguished human pluripotent stem cells from differentiated cells. The SS-DMRs defined in this experiment can be used as a signature for “stemness”. In addition, knowledge of the DNA methylation profile in human ES and iPS cells derived from different cell types is absolutely imperative and may allow us to screen for optimum iPS/ES cells and to validate and monitor iPS/ES cell derivatives for human therapeutic applications.

## Materials and Methods

### Human Cells

Human endometrium, bone marrow stroma, auricular cartilage, extra finger bone marrow, amnion, placental artery endothelium and menstrual blood cells were collected by scraping tissues from surgical specimens as a therapy, under signed informed consent, with ethical approval of the Institutional Review Board of the National Institute for Child Health and Development, Japan. Signed informed consent was obtained from donors, and the surgical specimens were irreversibly de-identified. All experiments handling human cells and tissues were performed in line with Tenets of the Declaration of Helsinki. Endometrium (UtE1104), bone marrow stroma (H4-1) [33], auricular cartilage (Mim1508E), extra finger bone marrow (Yub636BM), amnion (AM936EP), placental artery endothelium (PAE551) and menstrual blood cell (Edom22) [34] cell lines were independently established in our laboratory. H4-1, Mim1508E, Yub636BM, AM936EP, Edom22, and MRC-5 [35] cells were maintained in the POWEREDBY10 medium (MED SHIROTORI CO., Ltd, Tokyo, Japan). PAE551 were cultured in EGM-2MV BulletKit (Lonza, Walkersville, MD, USA) containing 5% FBS. Human induced pluripotent stem (iPS) cells were generated, via procedures described by Yamanaka and colleagues [2] with slight modification. Human iPS cell lines derived from MRC-5 were designated as MRC-iPS cells [13], also iPS cell lines from AM936EP were named as AM-iPS cells [12]. Human iPS cells were maintained in iPSellon medium (Cardio Incorporated, Osaka, Japan) supplemented with 10 ng/ml recombinant human basic fibroblast growth factor (bFGF, Wako Pure Chemical Industries, Ltd., Osaka, Japan). Frozen pellets of human ES cell (HUESCs) were kindly gifted from Drs. C. Cowan and T. Tenzan (Harvard Stem Cell Institute, Harvard University, Cambridge, MA).

### Illumina Infinium HumanMethylation27 BeadChip assay

Genomic DNA was extracted from cells using the QIAamp DNA Mini Kit (Qiagen). One microgram of genomic DNA from each sample was bisulfite-converted using EZ DNA Methylation-Gold kit (Zymo Research), according to the manufacturer's recommendations. Bisulfite-converted DNA was hybridized to the HumanMethylation27 BeadChip (Illumina inc.). Methylation levels of each CpG site were determined with fluorescent signals for methylated and unmethylated alleles. Methylated and unmethylated signals were used to compute a Beta value, which was a quantitative score of DNA methylation levels ranging from “0”, for completely unmethylated, to “1”, for completely methylated. On the HumanMethylation27 BeadChip, oligonucleotides for 27,578 CpG sites covering more than 14,000 genes are mounted, mostly selected from promoter regions. 26,956 (97.7%) out of the 27,578 CpG sites are set at promoters and 20,006 (72.5%) sites are set on CpG islands. CpG sites with ≥0.05 “Detection p value” (computed from the background based on negative controls) were eliminated from the data for further analysis, leaving 24,949 valid for use with the 16 samples tested.

### Analysis of DNA methylation data

To analyze DNA methylation data, we used the following web tools: TIGR MeV [36] (http://www.tm4.org/mev.html) for hierarchical clustering heat map, NIA Array [37] (http://lgsun.grc.nia.nih.gov/ANOVA/) for hierarchical clustering that classify DNA methylation data by similarity and for principal component analysis (PCA) that finds major component in data variability, DAVID Bioinformatics Resources [38] (http://david.abcc.ncifcrf.gov/home.jsp), PANTHER Classification System [39] (http://www.pantherdb.org/), WebGestalt [40] (WEB-based GEne SeT AnaLysis Toolkit) (http://bioinfo.vanderbilt.edu/webgestalt/) based on based on KEGG (Kyoto Encyclopedia of Genes and Genomes) database [41] (http://www.genome.jp/kegg/) for gene ontology analysis, the GEO database (http://www.ncbi.nlm.nih.gov/geo/) for surveying gene expression in human iPS/ES cells (accession no. GSE9832 [16] and GSE12583 [17]), and the UCSC Genome Browser website [42] (http://genome.ucsc.edu/).

### RT-PCR

RNA was extracted from cells using the RNeasy Plus Mini kit (Qiagen). An aliquot of total RNA was reverse transcribed using random hexamer primers. The cDNA template was amplified using specific primers for *SOX10*, *SOX15*, *PPP1R16B*, *SALL4*, *TDGF1*, *Sp100* and *GBP3*. Expression of glyceraldehyde-3-phosphate dehydrogenase (GAPDH) was used as a positive control. Primers used in this study are summarized in Table S6A.

### Quantitative combined bisulfite restriction analysis (COBRA) and bisulfite sequencing

To confirm DNA methylation state, bisulfite PCR-mediated restriction mapping (known as the COBRA method) was performed. Sodium bisulfite treatment of genomic DNA was carried out as described above. PCR amplification was performed using IMMOLASE™ DNA polymerase (Bioline Ltd; London, UK) and specific primers (Table S6B). After digestion with restriction enzymes, HpyCH4IV or Taq I, quantitative-COBRA coupled with the Shimadzu MCE®-202 MultiNA platform (Shimadzu, Japan) known as the Bio-COBRA method was carried out for quantitative DNA methylation level. Information of primers and restriction enzyme is summarized in Table S6B. To determine the methylation status of individual CpG in *SOX15*, *SALL4*, *Sp100* and *GBP3*, the PCR product was gel extracted and subcloned into pGEM T Easy vector (Promega, Madison, WI), and then sequenced. Methylation sites were visualized and quality control was carried out by the web-based tool, “QUMA” (http://quma.cdb.riken.jp/) [43].

## Supporting Information

Table S1 Frequency of methylation states in each cell line.
(0.04 MB PDF)

Table S2 A list of genes with SS-hyper-DMRs and SS-hypo-DMRs on KEGG Pathway.
(0.05 MB PDF)

Table S3 (A) DNA methylation states of 23 genes (26 CpG sites) in Table 3, (B) DNA methylation states of 43 genes (50 CpG sites) in Table S4.
(1.61 MB PDF)

Table S4 A list of 43 genes with SS-hyper-DMRs exhibiting ‘low’ expression in human iPS/ES cells.
(0.07 MB PDF)

Table S5 (A) DNA methylation states of DNA methyltransferases, (B) Histone methylation states of DNA methyltransferases, (C) DNA methylation states of marker genes in human iPS/ES cells.
(0.57 MB PDF)

Table S6 (A) primers used for RT-PCR, and (B) primers used for COBRA.
(0.52 MB PDF)

Data set S1 A list of overlapped aberrant DMSs.
(0.16 MB XLS)

Data set S2 A list of MRC-iPS specific aberrant DMSs.
(0.13 MB XLS)

Data set S3 A list of AM-iPS specific aberrant DMSs.
(0.25 MB XLS)

Data set S4 A list of SS-hyper-DMRs.
(0.61 MB XLS)

Data set S5 A list of SS-hypo-DMRs.
(0.10 MB XLS)
