# Supplementary material for: Defining Hypo-Methylated Regions of Stem Cell-Specific Promoters in Human iPS Cells Derived from Extra-Embryonic Amnions and Lung Fibroblasts
Source: PLoS One. 2010 Sep 27;5(9):e13017. doi: 10.1371/journal.pone.0013017 (PMC2946409; doi:10.1371/journal.pone.0013017)
Supplement: Table S1 — Frequency of methylation states in each cell line. (0.04 MB PDF) [file pone.0013017.s001.pdf]

Table S1. Frequency of methylation states in the 24,949 CpG sites.

	Methylation		
	Low	Middle	High
Cell ID	Score ≤ 0.3	0.3 < Score ≤ 0.7	0.7 < Score
MRC5	17563 (70.4%)	3053 (12.2%)	4333 (17.4%)
MRC-iPS-11	16504 (66.2%)	1676 (6.7%)	6769 (27.1%)
MRC-iPS-19	16497 (66.1%)	1839 (7.4%)	6613 (26.5%)
MRC-iPS-75	16433 (65.9%)	1834 (7.4%)	6682 (26.8%)
AM936EP	17654 (70.8%)	3171 (12.7%)	4124 (16.5%)
AM-iPS-3	16499 (66.1%)	3038 (12.2%)	5412 (21.7%)
AM-iPS-6	16547 (65.3%)	3001 (12.0%)	5401 (21.6%)
AM-iPS-8	16347 (66.5%)	2719 (10.9%)	5883 (23.6%)
UtE1104	17471 (70.0%)	2708 (10.9%)	4770 (19.1%)
H4-1	17275 (69.2%)	3186 (12.8%)	4488 (18.0%)
Mim1508E	18094 (72.5%)	2593 (10.4%)	4262 (17.1%)
Yub636BM	17787 (71.3%)	2728 (10.9%)	4434 (17.8%)
PAE551	17657 (70.8%)	3122 (12.5%)	4170 (16.7%)
Edom22	17560 (70.4%)	3123 (12.5%)	4266 (17.1%)
HUES3	16437 (65.9%)	1972 (7.9%)	6540 (26.2%)
HUES8	16342 (65.5%)	2007 (8.0%)	6600 (26.5%)
