# Supplementary material for: Defining Hypo-Methylated Regions of Stem Cell-Specific Promoters in Human iPS Cells Derived from Extra-Embryonic Amnions and Lung Fibroblasts
Source: PLoS One. 2010 Sep 27;5(9):e13017. doi: 10.1371/journal.pone.0013017 (PMC2946409; doi:10.1371/journal.pone.0013017)
Supplement: Table S2 — A list of genes with SS-hyper-DMRs and SS-hypo-DMRs on KEGG Pathway. (0.05 MB PDF) [file pone.0013017.s002.pdf]

Table S2

SS-hyper-DMRs

KEGG pathway	Gene number	Entrez Gene IDs
Cytokine-cytokine receptor interaction	22	10673 1436 1440 3082 3455 3553 3575 3590 3626 4055 50615 55504 56477 58985 6347 6362 6376 64806 7043 7293 8200 8764
MAPK signaling pathway	15	10000 10454 11184 23581 27091 2885 3316 3553 5319 5533 55970 5604 7043 8074 834
Neuroactive ligand-receptor interaction	13	114131 152 1908 2359 2562 2569 3356 4159 4160 4161 6755 9002 90226
Hematopoietic cell lineage	12	1436 1440 2208 3553 3575 3590 910 913 916 930 945 966
Focal adhesion	12	10000 1281 1293 2885 3082 330 3479 3691 5500 5604 5649 5829
Insulin signaling pathway	9	10000 1977 23265 2885 5260 5500 5604 805 808
Jak-STAT signaling pathway	9	10000 1440 2885 3455 3575 3590 50615 58985 6774
Toll-like receptor signaling pathway	8	10000 10454 114609 3455 3553 3929 7098 7099
Glioma	7	10000 1026 2885 3479 5604 805 808
Regulation of actin cytoskeleton	7	10109 3691 5500 55970 5604 5829 8074
Natural killer cell mediated cytotoxicity	6	2885 3455 3821 5533 5604 9437
Complement and coagulation cascades	6	5265 5345 714 715 716 966
VEGF signaling pathway	6	10000 3316 5319 5533 5604 5829
Axon guidance	6	10501 2042 29984 3983 5364 5533
Chronic myeloid leukemia	6	10000 1026 2885 5604 7043 861
Calcium signaling pathway	6	3356 5136 5260 5533 805 808
Glycolysis / Gluconeogenesis	5	130589 222 26330 5224 5238
Purine metabolism	5	203 27115 2974 5136 5433
Cell Communication	5	1281 1293 2705 3691 5649
Cell adhesion molecules (CAMs)	5	1003 4099 5133 80380 9369
Gap junction	5	1453 2885 2974 3356 5604
T cell receptor signaling pathway	5	10000 2885 5133 5533 916
Long-term potentiation	5	5500 5533 5604 805 808
GnRH signaling pathway	5	2885 5319 5604 805 808
Glycan structures - biosynthesis 2	4	2523 2525 54965 8707
Apoptosis	4	10000 330 3553 5533
ECM-receptor interaction	4	1281 1293 3691 5649
Pancreatic cancer	4	10000 5604 6774 7043
Long-term depression	4	2974 3479 5319 5604
Huntington's disease	4	2885 805 808 834
Colorectal cancer	4	10000 2885 5604 7043
Fc epsilon RI signaling pathway	4	10000 2885 5319 5604
Pyrimidine metabolism	3	10587 5433 83549
Leukocyte transendothelial migration	3	1003 29119 5829
Tyrosine metabolism	3	1638 222 8639
Phenylalanine metabolism	3	222 4353 8639
Tryptophan metabolism	3	11185 66002 8639
Glutathione metabolism	3	2687 2880 2949
Starch and sucrose metabolism	3	2990 5238 79132
Arachidonic acid metabolism	3	2687 2880 5319
Glycosphingolipid biosynthesis - lactoseries	3	2523 2525 8707
PPAR signaling pathway	3	1582 3158 6342
Phosphatidylinositol signaling system	3	5298 805 808
Cell cycle	3	1026 1111 7043
mTOR signaling pathway	3	10000 1977 3479
Wnt signaling pathway	3	23500 4316 5533
TGF-beta signaling pathway	3	3626 7043 8200
Tight junction	3	10000 29119 93643
B cell receptor signaling pathway	3	10000 5533 930
Glycine, serine and threonine metabolism	3	145226 189 8639

SS-hypo-DMRs

KEGG pathway	Gene number	Entrez Gene IDs
Neuroactive ligand-receptor interaction	3	51083 7068 7200
TGF-beta signaling pathway	2	1030 4087
Wnt signaling pathway	2	4087 81839
Calcium signaling pathway	2	2778 814
Cell cycle	2	1030 4087
Adherens junction	2	4087 5777
