# Supplementary material for: Defining Hypo-Methylated Regions of Stem Cell-Specific Promoters in Human iPS Cells Derived from Extra-Embryonic Amnions and Lung Fibroblasts
Source: PLoS One. 2010 Sep 27;5(9):e13017. doi: 10.1371/journal.pone.0013017 (PMC2946409; doi:10.1371/journal.pone.0013017)
Supplement: Table S3 — (A) DNA methylation states of 23 genes (26 CpG sites) in Table 3, (B) DNA methylation states of 43 genes (50 CpG sites) in Table S4. (1.61 MB PDF) [file pone.0013017.s003.pdf]

Table S3

A, DNA methylation states of 23 genes (26 CpG sites) in Table 3

TargetID	SYMBOL	HES-3	HES-8	AM-iPS-3	AM-iPS-6	AM-iPS-8	MRC-iPS-11	MRC-iPS-19	MRC-iPS-75	AM936EP	MRC5	UtE1104	H4-1	Mim1508E	Yub636BM	PL551Ar	Edom22	Distance to TSS
cg07337598	ANXA9	0.317	0.258	0.252	0.221	0.253	0.305	0.428	0.321	0.729	0.700	0.712	0.675	0.702	0.769	0.755	0.653	611
cg24183173	BCOR	0.018	0.018	0.040	0.017	0.022	0.000	0.000	0.000	0.676	0.964	0.686	0.744	0.672	0.969	0.930	0.631	141
cg21207436	C14orf115	0.075	0.071	0.041	0.034	0.047	0.043	0.056	0.048	0.434	0.265	0.423	0.510	0.487	0.433	0.369	0.610	104
cg22892904	CBX2	0.076	0.073	0.062	0.084	0.054	0.038	0.077	0.081	0.848	0.580	0.563	0.546	0.785	0.622	0.427	0.481	904
cg24754277	DAPK1	0.114	0.122	0.136	0.114	0.126	0.115	0.101	0.096	0.795	0.476	0.900	0.585	0.627	0.741	0.730	0.811	
cg21629895	DNMT3A	0.477	0.439	0.418	0.421	0.474	0.442	0.441	0.506	0.906	0.806	0.789	0.912	0.699	0.640	0.614	0.790	336
cg02932167	ECEL1	0.107	0.137	0.120	0.107	0.087	0.126	0.096	0.141	0.891	0.486	0.815	0.694	0.710	0.595	0.400	0.784	420
cg25431974	ECEL1	0.061	0.150	0.108	0.120	0.187	0.113	0.150	0.107	0.966	0.329	0.890	0.829	0.782	0.584	0.247	0.762	405
cg04515567	FOXH1	0.620	0.672	0.560	0.588	0.607	0.612	0.543	0.614	0.850	0.815	0.852	0.871	0.871	0.851	0.869	0.858	150
cg04464446	GAL	0.158	0.142	0.220	0.256	0.302	0.282	0.260	0.309	0.918	0.715	0.926	0.632	0.637	0.596	0.547	0.908	818
cg00943909	GNAS	0.060	0.100	0.126	0.145	0.078	0.039	0.025	0.031	0.664	0.320	0.646	0.668	0.595	0.624	0.038	0.668	
cg27661264	GNAS	0.041	0.062	0.035	0.050	0.016	0.025	0.027	0.036	0.437	0.200	0.443	0.465	0.357	0.442	0.046	0.452	
cg18741908	GPR160	0.063	0.083	0.085	0.059	0.051	0.093	0.052	0.058	0.427	0.535	0.495	0.436	0.607	0.294	0.355	0.581	115
cg20674521	KCNJ4	0.298	0.400	0.249	0.248	0.201	0.350	0.347	0.352	0.597	0.697	0.867	0.944	0.891	0.673	0.698	0.808	682
cg21129531	LRRC4	0.026	0.028	0.033	0.027	0.036	0.004	0.023	0.039	0.757	0.553	0.889	0.986	0.936	0.760	0.868	0.553	299
cg06144905	PIPOX	0.132	0.158	0.048	0.062	0.059	0.072	0.142	0.123	0.507	0.615	0.763	0.894	0.670	0.379	0.177	0.459	138
cg13083810	POU5F1	0.627	0.669	0.455	0.498	0.526	0.609	0.572	0.550	0.941	0.925	0.910	0.937	0.941	0.906	0.925	0.869	703
cg27377213	PPP1R16B	0.091	0.122	0.075	0.096	0.081	0.086	0.150	0.072	0.939	0.727	0.960	0.957	0.942	0.822	0.114	0.908	545
cg19580810	RAB25	0.118	0.094	0.038	0.052	0.044	0.042	0.059	0.049	0.709	0.812	0.726	0.703	0.685	0.528	0.774	0.686	200
cg09243900	RAB25	0.128	0.181	0.065	0.094	0.099	0.069	0.121	0.085	0.570	0.645	0.596	0.704	0.561	0.446	0.534	0.704	138
cg06303238	SALL4	0.000	0.014	0.030	0.035	0.025	0.000	0.000	0.000	0.934	0.806	0.903	0.970	0.729	0.372	0.678	0.498	55
cg06614002	SOX10	0.026	0.062	0.021	0.019	0.024	0.028	0.023	0.016	0.930	0.641	0.911	0.924	0.906	0.607	0.837	0.872	100
cg01029592	SOX15	0.112	0.188	0.150	0.187	0.192	0.203	0.169	0.191	0.828	0.746	0.711	0.578	0.562	0.759	0.689	0.661	35
cg10242476	TDGF1	0.096	0.110	0.192	0.185	0.184	0.150	0.127	0.121	0.406	0.198	0.470	0.624	0.448	0.444	0.176	0.334	151
cg20277416	TM7SF2	0.354	0.363	0.368	0.395	0.489	0.372	0.382	0.319	0.722	0.874	0.762	0.938	0.917	0.851	0.795	0.803	313
cg05656364	VAMP8	0.090	0.134	0.063	0.054	0.055	0.050	0.060	0.054	0.910	0.743	0.713	0.891	0.576	0.706	0.198	0.849	15

B, DNA methylation states of 33 genes (50 CpG sites) in Table S4

TargetID	SYMBOL	HES-3	HES-8	AM-iPS-3	AM-iPS-6	AM-iPS-8	MRC-iPS-11	MRC-iPS-19	MRC-iPS-75	AM936EP	MRC5	UtE1104	H4-1	Mim1508E	Yub636BM	PL551Ar	Edom22	Distance to TSS
cg16608652	B3GALT2	0.725	0.621	0.670	0.729	0.720	0.884	0.853	0.879	0.053	0.033	0.120	0.027	0.049	0.024	0.615	0.129	334
cg00725635	B3GALT2	0.297	0.333	0.597	0.498	0.611	0.555	0.629	0.460	0.095	0.009	0.000	0.016	0.003	0.000	0.138	0.120	82
cg14481222	BIRC3	0.442	0.334	0.581	0.540	0.607	0.666	0.585	0.829	0.064	0.111	0.069	0.096	0.047	0.054	0.071	0.065	220
cg15776355	C1R	0.649	0.706	0.604	0.661	0.682	0.639	0.660	0.647	0.220	0.050	0.064	0.121	0.076	0.048	0.083	0.103	693
cg05538432	C1S	0.691	0.660	0.524	0.522	0.565	0.812	0.657	0.735	0.126	0.176	0.165	0.142	0.172	0.070	0.213	0.174	266
cg13802966	CASP1	0.880	0.931	0.879	0.860	0.889	0.924	0.941	0.929	0.421	0.428	0.228	0.438	0.232	0.385	0.516	0.290	23
cg24453664	CD59	0.258	0.245	0.492	0.409	0.503	0.531	0.469	0.445	0.041	0.036	0.034	0.021	0.019	0.039	0.037	0.053	389
cg02189785	CLIC3	0.549	0.472	0.465	0.391	0.349	0.604	0.566	0.626	0.078	0.298	0.068	0.042	0.058	0.038	0.301	0.041	16
cg20802392	CTSK	0.519	0.599	0.643	0.621	0.545	0.776	0.759	0.816	0.055	0.320	0.017	0.009	0.008	0.002	0.623	0.044	128
cg24292612	DEFB1	0.875	0.866	0.874	0.868	0.881	0.921	0.881	0.903	0.037	0.135	0.083	0.276	0.635	0.333	0.791	0.230	57
cg05822532	ELN	0.520	0.473	0.682	0.665	0.777	0.632	0.599	0.632	0.044	0.117	0.148	0.000	0.008	0.007	0.057	0.032	13
cg24910675	ENG	0.446	0.456	0.599	0.534	0.627	0.625	0.501	0.513	0.042	0.063	0.029	0.025	0.055	0.053	0.040	0.037	67
cg07233761	ESM1	0.587	0.554	0.475	0.468	0.608	0.641	0.533	0.413	0.111	0.017	0.009	0.017	0.007	0.183	0.022	0.153	27
cg07354209	FAP	0.675	0.692	0.433	0.490	0.600	0.771	0.723	0.837	0.098	0.192	0.065	0.097	0.127	0.085	0.111	0.178	6
cg10503234	FSTL3	0.229	0.285	0.312	0.355	0.302	0.459	0.395	0.411	0.033	0.065	0.045	0.076	0.029	0.020	0.022	0.024	322
cg22074858	GBP3	0.871	0.936	0.761	0.744	0.758	0.957	0.933	0.914	0.072	0.121	0.281	0.159	0.521	0.050	0.072	0.080	114
cg15783800	HAK	0.924	0.961	0.857	0.855	0.895	0.960	0.943	0.957	0.173	0.013	0.014	0.515	0.007	0.025	0.822	0.212	404
cg08005849	HGF	0.565	0.562	0.532	0.500	0.551	0.640	0.583	0.639	0.072	0.116	0.062	0.083	0.155	0.082	0.175	0.072	53
cg04312209	IL7R	0.473	0.457	0.426	0.450	0.466	0.787	0.682	0.793	0.101	0.000	0.000	0.018	0.000	0.000	0.134	0.128	139
cg06130787	KLK10	0.741	0.699	0.759	0.746	0.776	0.842	0.834	0.894	0.084	0.061	0.248	0.331	0.148	0.115	0.149	0.075	268
cg11471401	KRT6A	0.778	0.823	0.737	0.719	0.732	0.922	0.869	0.857	0.210	0.488	0.275	0.254	0.112	0.085	0.459	0.093	1460
cg25620220	LOC221091	0.919	0.929	0.956	0.960	0.944	0.978	0.968	0.960	0.113	0.274	0.055	0.115	0.117	0.068	0.894	0.040	125
cg15538427	LOC221091	0.575	0.613	0.862	0.834	0.873	0.966	0.925	0.953	0.050	0.074	0.010	0.016	0.026	0.006	0.456	0.028	186
cg16983159	LOC340061	0.866	0.938	0.888	0.873	0.891	0.959	0.861	0.943	0.091	0.113	0.037	0.000	0.021	0.007	0.033	0.041	149
cg08569678	LY6K	0.419	0.212	0.480	0.336	0.405	0.420	0.374	0.303	0.048	0.019	0.038	0.045	0.028	0.020	0.035	0.052	134
cg12360736	MBNL1	0.967	0.953	0.776	0.804	0.847	0.946	0.910	0.956	0.216	0.083	0.077	0.315	0.155	0.281	0.063	0.257	40
cg24541550	MRVI1	0.750	0.760	0.802	0.778	0.854	0.864	0.823	0.818	0.020	0.166	0.136	0.119	0.124	0.038	0.844	0.041	175
cg14209518	NNMT	0.850	0.808	0.626	0.709	0.717	0.918	0.910	0.897	0.222	0.277	0.100	0.089	0.088	0.069	0.146	0.136	101
cg09632136	NNMT	0.668	0.587	0.675	0.602	0.667	0.856	0.817	0.737	0.083	0.086	0.053	0.039	0.042	0.035	0.045	0.083	874
cg12584889	NXF3	0.869	0.898	0.779	0.770	0.813	0.878	0.828	0.842	0.512	0.466	0.444	0.417	0.606	0.548	0.583	0.650	97
cg15149645	P8	0.934	0.926	0.887	0.886	0.903	0.951	0.932	0.917	0.067	0.673	0.010	0.002	0.113	0.000	0.739	0.075	289
cg05590982	P8	0.833	0.818	0.662	0.711	0.766	0.787	0.865	0.841	0.091	0.060	0.022	0.039	0.070	0.009	0.482	0.108	159
cg14440664	PDCD1LG2	0.903	0.912	0.765	0.783	0.793	0.932	0.905	0.903	0.042	0.000	0.008	0.000	0.000	0.000	0.031	0.045	928
cg07211259	PDCD1LG2	0.717	0.867	0.704	0.666	0.724	0.895	0.870	0.868	0.041	0.000	0.000	0.003	0.000	0.000	0.035	0.026	73
cg09462575	PI3	0.747	0.722	0.572	0.602	0.567	0.746	0.644	0.667	0.224	0.207	0.257	0.257	0.289	0.249	0.189	0.239	13
cg03242666	PMP22	0.674	0.672	0.646	0.668	0.687	0.780	0.728	0.733	0.060	0.237	0.081	0.099	0.128	0.069	0.720	0.085	74
cg08343834	PMP22	0.549	0.599	0.643	0.659	0.677	0.715	0.691	0.693	0.162	0.426	0.090	0.091	0.123	0.110	0.516	0.101	14
cg00563845	PXK	0.425	0.482	0.348	0.373	0.340	0.371	0.434	0.441	0.088	0.242	0.086	0.155	0.133	0.084	0.218	0.107	312
cg10007262	RELN	0.335	0.279	0.303	0.358	0.361	0.267	0.345	0.280	0.029	0.044	0.080	0.019	0.009	0.008	0.019	0.017	1257
cg13997435	S100A2	0.479	0.514	0.651	0.693	0.652	0.687	0.729	0.723	0.041	0.052	0.075	0.059	0.044	0.059	0.030	0.034	100
cg05706061	SLC31A2	0.697	0.774	0.462	0.502	0.537	0.638	0.591	0.642	0.117	0.228	0.131	0.335	0.159	0.102	0.103	0.109	327
cg08331960	SLC9A3R2	0.852	0.875	0.889	0.902	0.942	0.954	0.955	0.941	0.228	0.712	0.191	0.380	0.333	0.135	0.390	0.236	332
cg12966875	SLPI	0.940	0.957	0.811	0.831	0.843	0.953	0.936	0.942	0.058	0.298	0.338	0.222	0.259	0.621	0.336	0.049	540
cg23539753	SP100	0.864	0.828	0.870	0.842	0.848	0.841	0.885	0.926	0.024	0.155	0.230	0.145	0.112	0.012	0.036	0.041	283
cg05091653	SP100	0.846	0.845	0.766	0.778	0.805	0.839	0.827	0.856	0.052	0.202	0.064	0.117	0.262	0.076	0.073	0.082	493
cg06101324	SPRR1A	0.646	0.592	0.874	0.859	0.884	0.859	0.936	0.866	0.041	0.069	0.092	0.066	0.145	0.143	0.172	0.459	1014
cg24884084	SPRR1B	0.667	0.580	0.741	0.696	0.752	0.853	0.910	0.714	0.074	0.009	0.025	0.122	0.067	0.040	0.055	0.259	481
cg13590277	SYNPO	0.741	0.694	0.782	0.838	0.810	0.837	0.770	0.824	0.075	0.020	0.015	0.019	0.057	0.080	0.015	0.122	744
cg02905245	URB	0.341	0.402	0.587	0.544	0.568	0.631	0.613	0.577	0.071	0.034	0.030	0.027	0.033	0.033	0.056	0.068	325
cg04195127	WFDC1	0.446	0.440	0.715	0.598	0.706	0.924	0.834	0.834	0.054	0.030	0.028	0.035	0.017	0.028	0.073	0.082	318
