# Supplementary material for: Defining Hypo-Methylated Regions of Stem Cell-Specific Promoters in Human iPS Cells Derived from Extra-Embryonic Amnions and Lung Fibroblasts
Source: PLoS One. 2010 Sep 27;5(9):e13017. doi: 10.1371/journal.pone.0013017 (PMC2946409; doi:10.1371/journal.pone.0013017)
Supplement: Table S4 — A list of 43 genes with SS-hyper-DMRs exhibiting ‘low’ expression in human iPS/ES cells. (0.07 MB PDF) [file pone.0013017.s004.pdf]

Table S4. A list of 43 genes with SS-hyper-DMRs exhibiting 'low' expression in human iPS/ES cells.

TargetID	Gene name	Fold change of expression	DNA methylation level iPS/ES cells	Diff. cells
cg16608652	*B3GALT2, UDP-Gal:betaGlcNAc beta 1;3-galactosyltransferase 2*	0.1575	0.760 ±0.035	0.131 ±0.071
cg00725635	*B3GALT2, UDP-Gal:betaGlcNAc beta 1;3-galactosyltransferase 2*	0.1575	0.497 ±0.045	0.048 ±0.021
cg14481222	*BIRC3, baculoviral IAP repeat-containing protein 3*	0.1853	0.573 ±0.052	0.072 ±0.007
cg15776355	*C1R, complement component 1; r subcomponent*	0.1757	0.656 ±0.011	0.096 ±0.020
cg05538432	*C1S, complement component 1; s subcomponent*	0.0656	0.646 ±0.037	0.155 ±0.015
cg13802966	*CASP1, caspase 1 isoform delta*	0.0840	0.904 ±0.011	0.367 ±0.037
cg24453664	*CD59, CD59 antigen p18-20*	0.1792	0.419 ±0.039	0.035 ±0.004
cg02189785	*CLIC3, chloride intracellular channel 3*	0.1321	0.503 ±0.035	0.116 ±0.040
cg20802392	*CTSK, cathepsin K preproprotein*	0.1983	0.660 ±0.039	0.135 ±0.079
cg24292612	*DEFB1, defensin; beta 1 preproprotein*	0.1544	0.884 ±0.007	0.315 ±0.095
cg05822532	*ELN, elastin*	0.0111	0.623 ±0.033	0.052 ±0.019
cg24910675	*ENG, endoglin precursor*	0.1720	0.538 ±0.025	0.043 ±0.005
cg07233761	*ESM1, endothelial cell-specific molecule 1 precursor*	0.1915	0.535 ±0.028	0.065 ±0.026
cg07354209	*FAP, fibroblast activation protein; alpha subunit*	0.1307	0.653 ±0.049	0.119 ±0.016
cg10503234	*FSTL3, follistatin-like 3 glycoprotein*	0.1059	0.344 ±0.027	0.039 ±0.007
cg22074858	*GBP3, guanylate binding protein 3*	0.0179	0.859 ±0.032	0.170 ±0.057
cg15783800	*HAK, heart alpha-kinase*	0.0709	0.919 ±0.016	0.223 ±0.105
cg08005849	*HGF, hepatocyte growth factor isoform 2 precursor*	0.0759	0.572 ±0.017	0.102 ±0.015
cg04312209	*IL7R, interleukin 7 receptor precursor*	0.0640	0.567 ±0.056	0.048 ±0.022
cg06130787	*KLK10, kallikrein 10 precursor*	0.1181	0.786 ±0.023	0.151 ±0.033
cg11471401	*KRT6A, keratin 6A*	0.1435	0.805 ±0.026	0.247 ±0.056
cg25620220	*LOC221091, hypothetical protein*	0.1542	0.952 ±0.007	0.209 ±0.101
cg15538427	*LOC221091, hypothetical protein*	0.1542	0.825 ±0.053	0.083 ±0.054
cg16983159	*LOC340061, hypothetical protein*	0.0818	0.902 ±0.014	0.043 ±0.014
cg08569678	*LY6K, lymphocyte antigen 6 complex; locus K*	0.1084	0.369 ±0.030	0.036 ±0.004
cg12360736	*MBNL1, muscleblind-like 1 isoform a*	0.1966	0.895 ±0.027	0.181 ±0.035
cg24541550	*MRVI1, JAW1-related protein isoform b*	0.1069	0.806 ±0.015	0.186 ±0.096
cg14209518	*NNMT, nicotinamide N-methyltransferase*	0.0490	0.804 ±0.039	0.141 ±0.026
cg09632136	*NNMT, nicotinamide N-methyltransferase*	0.0490	0.701 ±0.034	0.058 ±0.008
cg12584889	*NXF3, nuclear RNA export factor 3*	0.1786	0.835 ±0.016	0.528 ±0.029
cg15149645	*P8, p8 protein (candidate of metastasis 1)*	0.0366	0.917 ±0.008	0.210 ±0.109
cg05590982	*P8, p8 protein (candidate of metastasis 1)*	0.0366	0.785 ±0.025	0.110 ±0.054
cg14440664	*PDCD1LG2, programmed cell death 1 ligand 2*	0.0245	0.862 ±0.024	0.016 ±0.007
cg07211259	*PDCD1LG2, programmed cell death 1 ligand 2*	0.0245	0.789 ±0.033	0.013 ±0.006
cg09462575	*PI3, elafin preproprotein*	0.0281	0.658 ±0.026	0.239 ±0.011
cg03242666	*PMP22 , peripheral myelin protein 22*	0.1302	0.698 ±0.016	0.185 ±0.079
cg08343834	*PMP22 , peripheral myelin protein 22*	0.1302	0.653 ±0.019	0.202 ±0.060

cg00563845	*PXK, PX domain containing serine/ threonine kinase*	0.1686	0.402 ±0.018	0.139 ±0.022
cg10007262	*RELN, reelin isoform a*	0.1788	0.316 ±0.013	0.028 ±0.008
cg13997435	*S100A2, S100 calcium binding protein A2*	0.0373	0.641 ±0.033	0.049 ±0.005
cg05706061	*SLC31A2, solute carrier family 31 (copper transporters); member 2*	0.1161	0.605 ±0.037	0.161 ±0.029
cg08331960	*SLC9A3R2, solute carrier family 9 isoform 3 regulator 2*	0.1492	0.914 ±0.014	0.326 ±0.064
cg12966875	*SLPI, secretory leukocyte peptidase inhibitor precursor*	0.1515	0.902 ±0.022	0.273 ±0.064
cg23539753	*SP100, nuclear antigen Sp100*	0.1127	0.863 ±0.011	0.095 ±0.028
cg05091653	*SP100, nuclear antigen Sp100*	0.1127	0.820 ±0.012	0.116 ±0.027
cg06101324	*SPRR1A, small proline-rich protein 1A*	0.0024	0.815 ±0.044	0.148 ±0.047
cg24884084	*SPRR1B, small proline-rich protein 1B*	0.0308	0.739 ±0.037	0.081 ±0.028
cg13590277	*SYNPO, synaptopodin*	0.0518	0.787 ±0.018	0.050 ±0.014
cg02905245	*URB, steroid-sensitive protein 1*	0.0431	0.533 ±0.037	0.044 ±0.006
cg04195127	*WFDC1, WAP four-disulfide core domain 1 precursor*	0.0287	0.686 ±0.064	0.043 ±0.008

Fold change of expression: Fold change of expression of the listed gene in human iPS/ES cells against the expression level in differentiated cells.
