# Supplementary material for: Defining Hypo-Methylated Regions of Stem Cell-Specific Promoters in Human iPS Cells Derived from Extra-Embryonic Amnions and Lung Fibroblasts
Source: PLoS One. 2010 Sep 27;5(9):e13017. doi: 10.1371/journal.pone.0013017 (PMC2946409; doi:10.1371/journal.pone.0013017)
Supplement: Table S5 — (A) DNA methylation states of DNA methyltransferases, (B) Histone methylation states of DNA methyltransferases, (C) DNA methylation states of marker genes in human iPS/ES cells. (0.57 MB PDF) [file pone.0013017.s005.pdf]

Table S5

A, DNA methylation states of DNA methyltransferases

TargetID	SYMBOL	HES-3	HES-8	AM-iPS-3	AM-iPS-6	AM-iPS-8	MRC-iPS-11	MRC-iPS-19	MRC-iPS-75	AM936EP	MRC5	UtE1104	H4-1	Mim1508E	Yub636BM	PL551Ar	Edom22	Distance to TSS
cg15043801	DNMT1	0.036	0.015	0.042	0.049	0.049	0.045	0.048	0.051	0.136	0.020	0.036	0.015	0.020	0.011	0.014	0.036	100
cg17445987	DNMT1	0.037	0.051	0.037	0.034	0.034	0.037	0.060	0.055	0.028	0.036	0.041	0.038	0.052	0.053	0.041	0.050	506
cg12066181	DNMT3A	0.027	0.055	0.034	0.033	0.046	0.038	0.066	0.091	0.044	0.038	0.031	0.034	0.048	0.005	0.041	0.060	171
cg21629895	DNMT3A	0.477	0.439	0.418	0.421	0.474	0.442	0.441	0.506	0.906	0.806	0.789	0.912	0.699	0.640	0.614	0.790	336
cg16523653	DNMT3B	0.021	0.024	0.010	0.012	0.012	0.020	0.023	0.018	0.007	0.009	0.025	0.023	0.023	0.024	0.021	0.020	203
cg17482740	DNMT3B	0.346	0.359	0.177	0.177	0.205	0.330	0.365	0.304	0.514	0.405	0.419	0.245	0.364	0.375	0.379	0.302	764
cg27076046	DNMT3L	0.105	0.105	0.227	0.160	0.179	0.210	0.196	0.209	0.108	0.301	0.579	0.127	0.229	0.106	0.311	0.216	1429

B, Histone methylation states of DNMT3B and DNMT3L

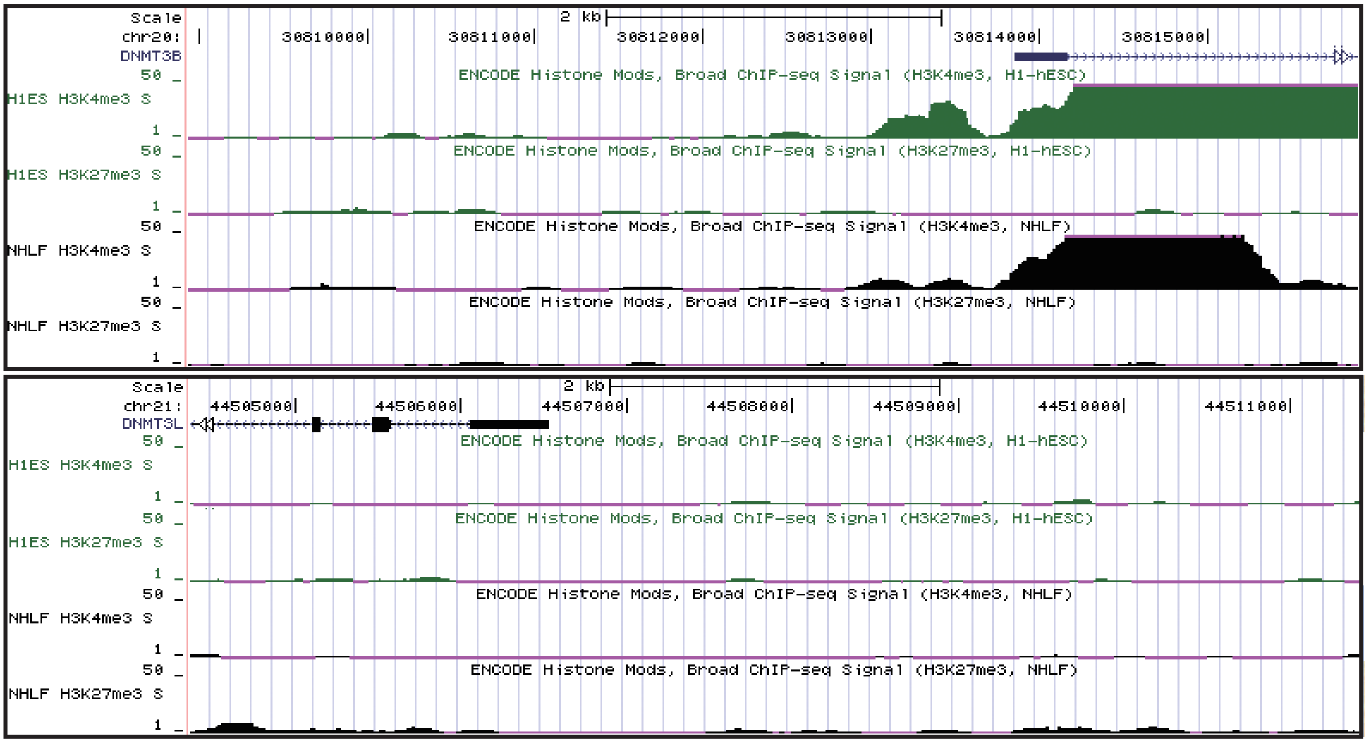

C, DNA methylation states of marker genes in human iPS/ES cells

TargetID	SYMBOL	HES-3	HES-8	AM-iPS-3	AM-iPS-6	AM-iPS-8	MRC-iPS-11	MRC-iPS-19	MRC-iPS-75	AM936EP	MRC5	UtE1104	H4-1	Mim1508E	Yub636BM	PL551Ar	Edom22	Distance to TSS
cg11620873	CD9	0.046	0.019	0.025	0.028	0.026	0.042	0.050	0.047	0.026	0.049	0.031	0.028	0.027	0.042	0.029	0.026	40
cg08519905	CD9	0.050	0.011	0.092	0.093	0.107	0.075	0.093	0.056	0.063	0.383	0.034	0.477	0.110	0.138	0.226	0.062	781
cg19831575	FGF4	0.016	0.010	0.012	0.010	0.010	0.001	0.020	0.017	0.012	0.051	0.508	0.259	0.026	0.018	0.018	0.034	81
cg15940569	GABRB3	0.343	0.424	0.300	0.342	0.368	0.340	0.379	0.355	0.067	0.131	0.103	0.058	0.099	0.100	0.073	0.096	429
cg17740645	GRB7	0.008	0.000	0.017	0.014	0.018	0.000	0.002	0.000	0.042	0.217	0.024	0.072	0.179	0.030	0.044	0.025	226
cg14967066	IFITM1	0.133	0.148	0.100	0.108	0.176	0.258	0.195	0.150	0.042	0.802	0.081	0.850	0.500	0.519	0.430	0.035	488
cg04377282	NODAL	0.124	0.096	0.052	0.049	0.056	0.074	0.104	0.054	0.375	0.229	0.451	0.120	0.259	0.118	0.111	0.531	6
cg00186141	PODXL	0.019	0.031	0.021	0.016	0.023	0.018	0.041	0.023	0.016	0.019	0.025	0.023	0.016	0.019	0.018	0.019	400
cg16488098	PODXL	0.000	0.000	0.056	0.017	0.031	0.000	0.000	0.000	0.282	0.000	0.196	0.011	0.064	0.025	0.032	0.342	928
cg01340005	SOX2	0.002	0.008	0.014	0.017	0.019	0.009	0.001	0.000	0.046	0.060	0.019	0.016	0.021	0.004	0.026	0.026	262
cg15105987	SOX2	0.077	0.081	0.507	0.322	0.382	0.488	0.466	0.459	0.080	0.239	0.212	0.335	0.177	0.096	0.114	0.235	595
cg02545192	TERT	0.034	0.015	0.085	0.055	0.046	0.043	0.052	0.044	0.019	0.109	0.037	0.027	0.056	0.017	0.024	0.022	430
cg15915418	TLE1	0.115	0.152	0.481	0.415	0.416	0.129	0.223	0.150	0.487	0.126	0.620	0.621	0.596	0.049	0.056	0.550	5
cg03755123	UTF1	0.049	0.060	0.175	0.176	0.179	0.152	0.162	0.122	0.064	0.103	0.079	0.067	0.052	0.036	0.104	0.077	270
cg09053680	UTF1	0.021	0.083	0.008	0.011	0.010	0.005	0.013	0.035	0.008	0.080	0.016	0.019	0.015	0.013	0.023	0.021	336
