# Supplementary material for: Defining Hypo-Methylated Regions of Stem Cell-Specific Promoters in Human iPS Cells Derived from Extra-Embryonic Amnions and Lung Fibroblasts
Source: PLoS One. 2010 Sep 27;5(9):e13017. doi: 10.1371/journal.pone.0013017 (PMC2946409; doi:10.1371/journal.pone.0013017)
Supplement: Table S6 — (A) primers used for RT-PCR, and (B) primers used for COBRA. (0.52 MB PDF) [file pone.0013017.s006.pdf]

Table S6

A, Primers used for RT-PCR

SYMBOL	Forward primer (5' to 3')	Reverse primer (5' to 3')	PCR size
SOX15	CAGAGGCTTTGGGTACAGAC	AGCAAGGGGAGGGTTGTATG	199
SALL4	GAAAACGGTTCCGTGGAGAG	ACGAGAAGTTCTTCCCACACC	394
TDGF1	TCCTTCTACGGACGGAACTG	AGAAATGCCTGAGGAAAGCA	140
PPP1R16B	AGCAGCTGAAGAAATGGGCAC	ACGAGGATCTTCACCAGGTTG	367
SOX10	AACGGCGCCAGCAAAAGCAA	TGGTACTTGTAGTCCGGGTG	239
GBP3	AACCCCTCACACCAGATGAG	CAGATGGTCCACATCCTTGA	461

SYMBOL	Forward primer (5' to 3')	Reverse primer (5' to 3')	PCR size
SP100	ACCGAGAAGTGAGCCTGTGA	GCTGTGATCCTGACCCTCTTC	398
DNMT1	ATTCTGATGGATCCCAGTCCC	GTCCTTCTCCCTGGTAGAATG	378
DNMT3A	GCCAAAACTGCAAGAACTGC	CCTTTGGAGGGTCAAATTCC	324
DNMT3B	AATCCTGGAGGCTATCCGCAC	GTCAGAGCCATCCCCATCTTC	142
DNMT3L	ATGTGGTTGATGTCACAGAC	GACAGCATTCTGCAAGGATC	305
GAPDH	GCTCAGACACCATGGGGAAGGT	GTGGTGCAGGAGGCATTGCTGA	474

B, Primers used for COBRA

SYMBOL	SYNONYM	Probe ID	Distance to TSS	CpG island	PCR region	PCR size	RE	Forward primer (5' to 3')	Reverse primer (5' to 3')
GBP3	FLJ10961	cg22074858	114	FALSE	−48 to +344	392	HpyCH4IV	gaaattgTatttatggttaTtgagtT	cctAtAtctcacatcaAActcaActA
LYST	CHS; CHS1;	cg12167564	94	FALSE	−231 to +118	349	HpyCH4IV	TaaagTTaaaaggtTattgggatggT	taAttttatAtcctccaaAAactAcaA
SP100	FLJ00340	cg23539753	283	FALSE	−5 to +334	339	HpyCH4IV	gTtgtgtattgTaTaaaTaagtggaT	tAAAcctcaAaAcctAAcctctAaAc
FOXP2	SPCH1;	cg05232889	143	FALSE	−343 to −8	335	HpyCH4IV	gtgtaTtTaTagtagtgtaaataTtgT	ctctaAttcatttcaatAaActacaA
GATAD2B	P66beta;	cg01402255	125	FALSE	−276 to +46	322	HpyCH4IV	agTagtTTtgttgtTtTTatgaggT	ttaaAAAactttattctAatcttacttA
ZFP37	FLJ38524;	cg03454353	368	TRUE	−539 to −145	394	HpyCH4IV	aTtaTaggaTtgaTTtgggTTtgaT	tatttctccaaAtActtAattattctA
PPP1R16B	TIMAP;	cg27377213	545	TRUE	−778 to −383	395	HpyCH4IV	taaagaaaggTtggtgTaTagagtgT	actcaAAAttcctaActtAaaAAcctA
PTPN6	HCP; SHP1;	cg04956511	268	TRUE	−546 to −135	411	HpyCH4IV	TtgTagtgTTattggTTtggTaggT	tAAaAaAaacaAatacacacttAtccaA
SOX10	DOM; WS4;	cg06614002	100	FALSE	−255 to +234	489	HpyCH4IV	TtTTaTTtTaTagTagggtTTTaggT	caAactaaAcccaaAataaAcctcaA
SOX15	SOX20; SOX26;	cg01029592	35	FALSE	−49 to +259	308	Taq I	aagaggtgttTtgaggTTtaTtgaT	AacaaacaAAacacctAAAtttacct
SALL4	DRRS; HSAL4;	cg06303238	55	TRUE	−66 to +247	313	Taq I	ggTTaatTagTtgtTagggTtTatga	ccccaAccccactcacccaActcc
TDGF1	CR; CRGF; CRIPTO;	cg27371741	55	TRUE	−143 to +183	326	Taq I	gTaaTtaatgatagagatattagggT	caAccaaaAAaaaacattcatctccta

TSS, Transcription stat site. CpG island, The loci is located in a CpG island (TRUE) or not (FALSE). RE, Restriction enzyme used for COBRA. Upper cases in primer sequences represent converted nucleotide for bisulfite PCR.
